# Prone versus Barts “flank-free” modified supine percutaneous nephrolithotomy: a match-pair analysis

**DOI:** 10.3906/sag-2011-21

**Published:** 2021-06-28

**Authors:** Uygar MİÇOOĞULLARI, Davut KAMACI, Mehmet YILDIZHAN, Furkan UMUT KILIÇ, Taha ÇETİN, Özer Ural ÇAKICI, Murat KESKE, Mehmet YİĞİT YALÇIN, Arslan ARDIÇOĞLU

**Affiliations:** 1 Department of Urology, University of Health Sciences, Tepecik Education and Research Hospital, İzmir Turkey; 2 Department of Urology, Ankara City Hospital, Ankara Turkey; 3 Department of Urology, Ankara Yildirim Beyazit University, School of Medicine affiliated with Ministry of Health Ankara City Hospital, Ankara Turkey; 4 Department of Urology, Ankara Medical Park Hospital, Ankara Turkey; 5 Department of Urology, Kayseri City Hospital, Kayseri Turkey

**Keywords:** Percutaneous nephrolithotomy, prone, supine, Barts “flank-free” modified supine position, stone-free rate, kidney stone

## Abstract

**Background/aim:**

In this study, we aimed to compare the results of prone and Barts “flank-free” modified supine percutaneous nephrolithotomy (PCNL) operations in our clinic.

**Materials and methods:**

The data from patients that underwent Barts “flank-free” modified supine PCNL (BS-PCNL) (n = 52) between June 2018 and July 2020 and prone PCNL (P-PCNL) (n = 286) between April 2014 and June 2018 were retrospectively evaluated. Of those 286 patients, 104 patients whose sex, age, body mass index, American Society of Anesthesiology score, stone localization, stone size, and hydronephrosis matched the BS-PCNL group in a 1:2 ratio were included in the study. The groups were compared in terms of intraoperative outcome, complication rates, and stone-free rates.

**Results:**

The mean age of all patients (58 females, 98 males) included in the study was 41.8 ± 15.2 years, and the mean body mass index (BMI) was 24.7 ± 2.9 kg/m2. The mean operation time was significantly shorter in the BS-PCNL group than in the P-PCNL group (80.2 ± 15.1 min vs. 92.4 ± 22.7 min and p = 0.01). There was no significant difference between the two groups in terms of fluoroscopy time, intraoperative complications, postoperative complications, and stone-free rates.

**Conclusion:**

Our study shows that BS-PCNL is an effective and safe method that significantly reduces the operation time and should be considered as one of the primary treatment options for patients scheduled for PCNL.

## 1. Introduction

According to current guidelines, the recommended treatment method for kidney stones larger than 20 mm is percutaneous nephrolithotomy (PCNL)The European Association of Urology Guidelines on Urolithiasis (2020). EAU Guidelines [online]. Website https://uroweb.org/wp-content/uploads/EAU-Guidelines-on-Urolithiasis-2020.pdf [accessed August 11, 2020]. [1]. PCNL was first described by Fernstrom and Johansson in 1976 [2]. Prone PCNL (P-PCNL) is the traditional position. Supine PCNL (S-PCNL) was first introduced in 1987 by Valdivia et al. [3]. Supine positions and modifications gained even more popularity after simultaneous retrograde approaches in S-PCNL were described by Ibarluzea et al. [4].

The prone position provides better pelvicalyceal imaging and a wider working area [5]. However, the supine position provides more comfort, lower renal pelvic pressure, higher lung ventilation pressure for the patient, easier respiratory system intervention for the anesthetist, and allows for simultaneous retrograde intrarenal surgery, as well as more comfortable anterior calyx access [6,7]. The results of studies comparing S-PCNL and P-PCNL are contradictory. Although various supine PCNL positions such as complete supine [8], Valdivia [9], Galdakao modified Valdivia [4], Barts modified Valdivia position [10], and Barts “flank-free” modified position [11] have been described, there still is no consensus on an ideal supine position.

The purpose of this study was to analyze and compare the data of the patients that underwent prone PCNL (P-PCNL) and Barts “flank-free” modified supine PCNL (BS-PCNL) [11].

## 2. Materials and methods

Our study was approved by the ethics committee of the Ankara Yildirim Beyazit University, School of Medicine (Institutional Review Board approval number: 26379996/58). All study participants signed the informed consent forms. The data of 52 patients that underwent BS-PCNL between June 2018 and July 2020 and the data of 286 patients that underwent P-PCNL between April 2014 and June 2018 were retrospectively reviewed. Of the 286 P-PCNL patients, 104 patients whose sex, age, body mass index (BMI), American Anesthesiology Association (ASA) score, stone localization, stone size, and hydronephrosis matched the BS-PCNL group in a 1:2 ratio were included in the study. Patients that underwent PCNL due to stones larger than 2 cm were included in the study. Before the study, serum biochemistry (creatinine, blood urea nitrogen, sodium, potassium, glomerular filtration value (GFR)), complete blood count, bleeding-clotting time, complete urinalysis, and urine culture were examined in all patients. All patients with positive growth in the urinary culture were treated with antibiotics suitable for the antibiogram result and underwent procedures only after their urine was proven to be sterile. Prior to the PCNL procedure, all patients were evaluated with low-dose non-contrast computed tomography (NCCT) for stone size, stone localization, stone density (Hounsefield unit), location of the colon, kidney parenchymal structure, kidney calyx anatomy, and entry tract. The longest diameter (millimeters) in the NCTT was used when recording stone sizes.

Two groups were compared in terms of demographic data (age, sex, previous surgery, ASA score, and BMI), stone properties (size, localization, hydronephrosis, opacity), surgical data (side, operation time, fluoroscopy time, access number, double J stent placement, nephrostomy placement, transfusion, and complications), and postoperative data (hospital length of stay, hemoglobin drop, transfusion, stone-free rate, and complications). Stone-free rates were evaluated as follows: stone-free after the first access (patients whose fluoroscopic imaging and visual examination indicated complete stone-free state after the first access in a single operation), stone-free after PCNL (patients that required second access during the same operation and thought to have reached complete stone-free state via visualization and fluoroscopic imaging after the second access), and overall stone-free rates (stone-free rates assessed by NCCT in the first postoperative month in both groups). Total surgery time was defined as the time between the initiation of anesthesia and the completion of the PCNL procedure. Stone-free state was defined as stone sizes of <3 mm. Intraoperative complications were evaluated according to the modified Stava classification system, while the postoperative complications were evaluated according to the modified Clavien–Dindo classification system [12,13].

### 2.1. Percutaneous nephrolithotomy procedures

All supine and prone procedures were performed by three experienced urologists at the tertiary referral center and all PCNL procedures were performed under general anesthesia. Barts “flank-free” modified supine position was used for the supine PCNL procedure [11]. The Barts “flank-free” modified supine position was chosen because of better patient comfort and lesser torso rotation compared with the Valdivia, Galdakao modified and the Barts modified Valdivia positions [11]. During this procedure, gel pads were placed under the ipsilateral rib cage and pelvis to ensure a 15° tilt of the ipsilateral flank at the supine position. The ipsilateral arm was outstretched to the contralateral side across the chest. The ipsilateral leg was extended, while the contralateral leg was held in the lithotomy position (Figure). A ureter catheter (5F) was inserted in the same position. In P-PCNL, the ureter catheter was inserted in the lithotomy position, then the patient was turned into a prone position. All calyx entries were performed under fluoroscopy. The puncture was performed with an 18-gauge percutaneous access needle. In both techniques, entry was performed from the posterior of the posterior axillary line. Entry tracts were dilated up to 28F. Then a 24F nephroscope (Karl Storz, Tuttlingen, Germany) was entered with amplatz. The stones were fragmented using a pneumatic lithotripter (Elmed, Ankara, Turkey). After the PCNL procedure was completed, a nephrostomy tube was placed based on the surgeon’s preference. Antegrade pyelography was performed to control for contrast extravasation or colon injury. In cases with residual stones or complications (e.g. renal pelvis injury), a double J (4.8F, 28 cm) stent (DJS) was placed in the ureter. In addition, in case of any complications that developed during and after PCNL, bleeding amount, and erythrocyte transfusion rates were recorded. On the first postoperative day, direct urinary system graphy (DUSG) was performed to assess the residual stone status in all patients and a chest radiograph was performed in patients that had a possibility of pleural injury. All patients were discharged after the nephrostomy catheters were removed. The overall stone-free status of the patients with DJS was assessed with noncontrast CT at the postoperative first month, and their DJSs were removed if appropriate.

**Figure:**
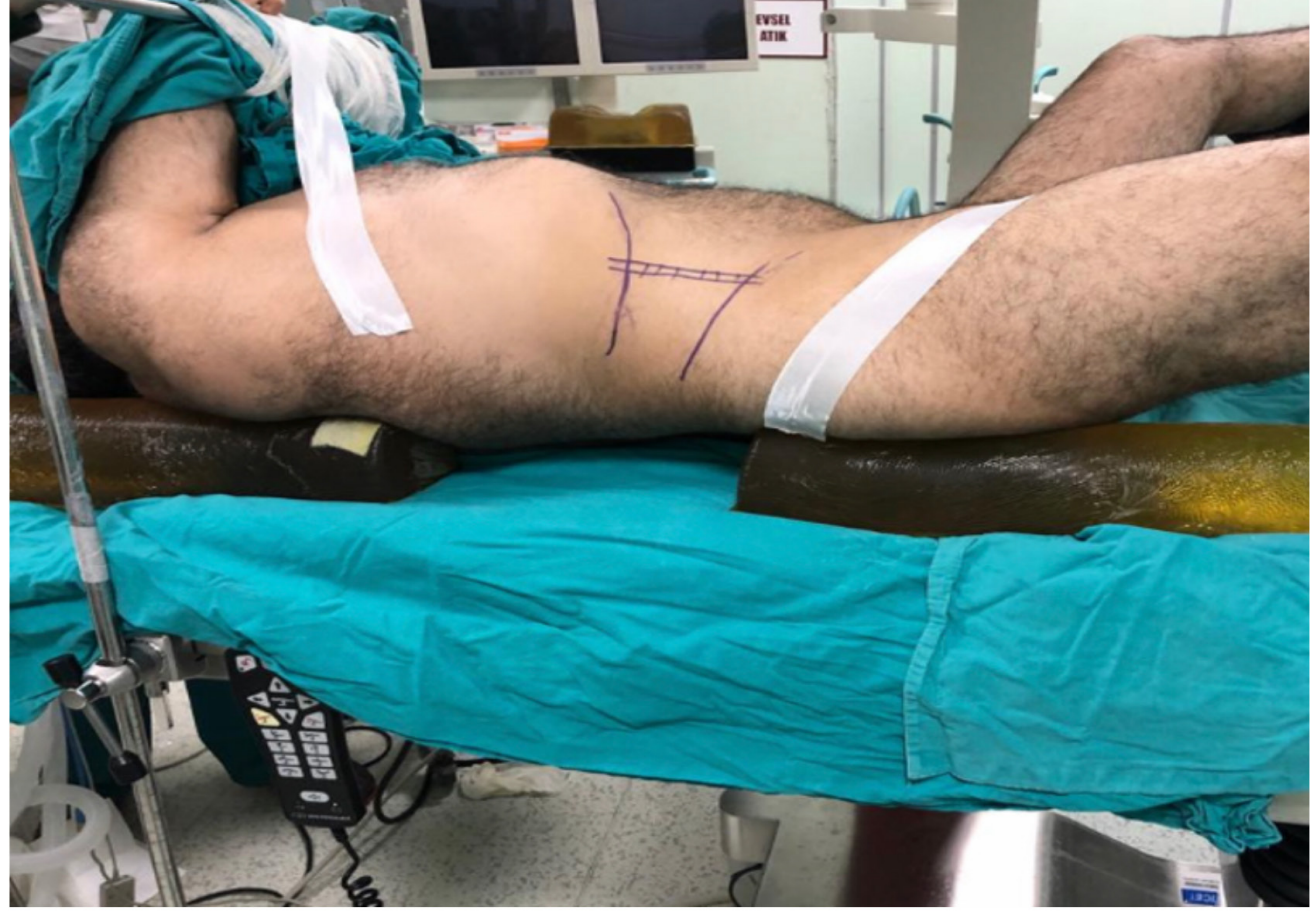
Barts “flank-free” modified supine position. Gel pad 1 is placed under the rib cage and gel pad 2 is under the ipsilateral pelvis.

### 2.2. Statistical analysis

SPSS v. 22.0 (Chicago, IL, USA) version for IBM was used for statistical analysis. Descriptive statistics of the groups were calculated. The Kolmogorov–Smirnov test was used to test if the variables showed a normal distribution. The variables fitting to the normal distribution were evaluated by the Student-t test and the ones that did not were evaluated by the Mann–Whitney U test. Besides, Chi-square and Fisher exact tests were used to evaluate categorical data. A p-value below 0.05 was considered significant.

## 3. Results

The mean age of all our patients (58 females, 98 males) was 41.8 ± 15.2 years and their mean body mass index (BMI) was 24.7 ± 2.9 kg/m2. While 44.2% of the stones were located in the renal pelvis, 42.3% were in the lower calyx, 6.4% were in the middle calyx, 2.6% were in the upper calyx, 2.6% were staghorn shaped, and 1.9% were located within multiple calyces. The mean stone size was 32.5 ± 7.9 mm. The mean ages of patients in the BS-PCNL and P-PCNL groups were 43.9 ± 16.2 and 40.8 ± 14.6, (p = 0.24), the sex distribution (F/M) was 20/32 and 38/66 (p = 0.81), while the mean BMIs were 24.4 ± 2.9 and 24.8 ± 2.9, respectively (p = 0.35) (Table 1). There was no significant difference between the groups in terms of age, sex distribution, BMI, stone size, ASA score, history of extracorporeal shock wave lithotripsy (ESWL) or other surgeries, stone opacity, stone density, stone localization, and preoperative hydronephrosis features. Stone characteristics and demographic data are summarized in Table 1.

**Table 1:** Demographic data and stone characteristics.

	BS-PCNL (n = 52)	P-PCNL (n = 104)	p
Sex (female/male)	20/32	38/66	0.81a
BMI	24.4 ± 2.9	24.8 ± 2.9	0.35b
Age	43.9 ± 16.2	40.8 ± 14.6	0.24b
ASA score123	36 (69.2%)14 (26.9%)2 (3.8%)	66 (63.5%)34 (32.7%)4 (3.8%)	0.75a
Previous ESWL/surgeryESWLURSPCNLOpen surgery	2 (3.8%)3 (5.8%)4 (7.7%)0	3 (2.9%)4 (3.8%)9 (8.7%)0	0.93a
Stone opacity (opaque/nonopaque).	48/4	93/11	0.56a
Stone density (HU), median (min-maks)	1180 (690–2080)	1130 (610–1980)	0.5c
Stone localization Lower calyxMiddle calyxUpper calyxPelvisMultiple calycesStaghorn-shaped	19 (36.5%)4 (7.7%)2 (3.8%)25 (48.1)1 (1.9%)1 (1.9%)	47 (45.2%)6 (5.8%)2 (1.9%)44 (42.3%)2 (1.9%)3 (2.9%)	0.88a
Stone size (mm)	32.1 ± 7.3	32.7 ± 8.2	0.38b
Hydronephrosis (no/mild/severe)	24/24/4	46/51/7	0.93a

Twenty-six patients in the P-PCNL group and 9 in the BS-PCNL group required second access during the same surgery due to residual stones. A complete stone-free state could not be achieved in 2 out of 9 patients in the BS-PCNL group even after the second access and simultaneous endoscopic combined intrarenal surgery (ECIRS) was performed in these patients.

The mean operation time was significantly shorter in the BS-PCNL group compared to the P-PCNL group (80.2 ± 15.1 min vs. 92.4 ± 22.7 min, p = 0.01). There was no significant difference between the two groups in terms of fluoroscopy period (p = 0.31) and intraoperative complications classified according to Satava classification (p = 0.49). In both groups totally, grade 1 complications were observed in 28 patients (BS-PCNL = 12, P-PCNL = 16) and grade 2a complications in 10 patients (BS-PCNL = 3, P-PCNL = 7) according to Satava classification. Operative data are summarized in Table 2.

**Table 2:** Operative data.

	BS-PCNL (n = 52)	P-PCNL (n = 104)	p
Operation side (right/left)	21/31	46/58	0.64a
Operation time (min)	80.2 ± 15.1	92.4 ± 22.7	0.001a
Fluoroscopy time (min)	3.53 ± 1.4	3.4 ± 1.2	0.31b
Double-J stent placement	3 (5.8%)	8 (7.7%)	0.75a
Nephrostomy placement	49 (94.2%)	96 (92.3%)	0.75a
Access poleLowerMiddleUpperMultiple	45 (86.5%)3 (5.8%)2 (3.8)2 (3.8%)	92 (88.5%)88 (7.7%)2 (1.9%)2 (1.9%)	0.75a
Entry localizationAbove 11th ribAbove 12th ribSubcostal	1 (1.9%)2 (3.8%)49 (94.2%)	3 (2.9%)5 (4.8%)96 (92.3%)	0.9a
Intraoperative complication Satava grade 1Satava grade 2	12 (23.1%)3 (5.8%)	16 (15.4%)7 (6.7%)	0.49a

Postoperative complications were classified according to Clavien–Dindo classification and the patient outcomes are summarized in Table 3. There was no significant difference between the two groups in terms of postoperative complications. In total, 17 patients had grade 1, 10 patients had grade 2, and 3 patients had grade 3a complications. When the stone-free rates after the PCNL procedure alone were evaluated, it was found to be proportionally higher in the P-PCNL group compared to the BS-PCNL group, however, this difference was not significant (prone: 94.2%, supine: 88.5%). When combined with ECIRS, the stone-free rate in the BS-PCNL group increased, but the difference was still not significant (prone: 94.2%, supine: 96.2%).

**Table 3:** Post-operative data.

	BS-PCNL (n = 52)	P-PCNL (n = 104)	p
Clavien–Dindo classificationGrade 1FeverSerum creatinine elevation Grade 2Blood transfusionUrinary tract infectionGrade 3aDouble-J stent placement for urine leakageAngioembolization	5 (9.6%)413 (5.8%)211 (1.9%)10	12 (11.5%)847 (6.7%)522 (1.9%)11	0.83a
Hematocrit drop (gr/dL)	3.2 ± 2.8	2.9 ± 2.6	0.31b
Hospital length of stay (day)	1.44 ± 0.77	1.36 ± 0.68	0.52b
Nephrostomy duration(day)	1.26 ± 0.56	1.25 ± 0.69	0.88b
Stone-free after the first access	43 (82.7%)	77 (74%)	0.31a
Stone-free after PCNL alone	46 (88.5%)	98 (94.2%)	0.21a
Overall stone-free rates	48 (92.3%)	98 (94.2%)	0.72a

## 4. Discussion 

In recent years, PCNL has become a gold standard for the treatment of kidney stones larger than 20 mm or stones that are complex in nature [1]. Although prone position was preferred in PCNL at first, over the years, supine or modified supine techniques have started to gain popularity [4,8–11]. In two major meta-analyzes; pooled data showed that PCNL in supine position could significantly reduce the operative time compared to the prone position [14,15]. Studies have reported similar stone-free rates, hospitalization, and complication rates in both positions [14]. 

While some recent meta-analyses state that there is no significant difference between S-PCNL and P-PCNL in terms of stone-free rates, there are other studies that claim the opposite [16–18]. In the multi-center Clinical Research Office of the Endourological Society (CROES) study, higher stone-free rates were reported after the P-PCNL operation compared to S-PCNL (59% vs. 48%, p < 0.001) [16]. Although the number of clinics participating in the CROES study was quite high (96 centers), the number of patients per clinic was relatively low (27 patients on average) [16]. This leads us to question the S-PCNL experience of the relevant centers. In addition, the difference in the level of experience of surgeons performing PCNL procedures should be taken into account. In another study comparing these two methods in staghorn stones by Gokce et al., it was emphasized that both PCNL techniques had similar stone-free rates (64% in the S-PCNL group and 60% in the P-PCNL group, p = 0.72) [17]. The striking feature of their study was that all cases were performed by one experienced surgeon. In another large-scale meta-analysis, data of 6881 patients were examined and significantly higher stone-free rates were reported in P-PCNL patients (77% vs. 74%, p < 0.001) [18]. In our study, the evaluation of stone-free rates showed no significant difference between the groups. We think that the use of Barts “flank-free” modified supine position and experience with prone PCNL contributed to this success. 

In a recent randomized controlled trial, the mean duration of surgery in patients undergoing P-PCNL was significantly longer than patients undergoing S-PCNL (111 min vs. 86 min) [19]. Moreover, in the review of 13 publications published by Yuan et al., the duration of surgery was shorter in patients that underwent S-PCNL [20]. However, in another meta-analysis compiling 20 studies, it was reported that P-PCNL does not prolong the total surgery time [21]. In our study, the mean duration of surgery was significantly shorter in the BS-PCNL group compared to the P-PCNL group, which was in line with most of the available publications.

Prone position restricts ventilation of the lungs and thus causes problems with oxygen saturation [6,7]. In our study, none of the patients had any complications related to oxygen saturation that changed the course of the operation. This might be because the majority of patients had ASA 1 or 2 scores, or because small restrictions might have gone unnoticed. Sharma et al. reported that while the probability of seeing retrocolon on the computed tomography taken in the supine position is 2%, that risk increases to 6.8% in the prone position [22]. In current clinical studies, the risk of colon injury is reported to be statistically similar in both positions (3.4% and 3.3% in the supine and prone positions, respectively, p = 0.958) [23]. In our study, no damage in the intestines, spleen, or liver were observed. Moreover, there was no significant difference between the groups in terms of intraoperative and postoperative complications according to Satava and Clavien classifications, respectively. The meta-analysis by Liu et al. also did not find any significant difference in complication rates between their modified supine and prone cohorts [24].

Unlike the prone position, the supine position bypasses the need for repositioning the patient, is more suitable for retrograde intrarenal surgery (RIRS), and prevents stress to the lungs and heart [6,7]. Despite our relatively low S-PCNL experience, our S-PCNL results were similar to P-PCNL, which is a sign of the reliability of S-PCNL. We think that the use of Barts “flank-free” modified supine position contributed to this success. 

The efficiency of ECIRS, simplicity of puncture during fluoroscopy, and the possibility of making and dilating multiple punctures vary in different supine position modifications. Although fluoroscopy-guided puncture of the renal tissue is not complicated when the patient is in the complete supine position, it may become challenging with increasing rotation of the patient’s torso. Therefore, Valdivia and modified Valdivia positions that have a 30° tilt and Barts modified Valdivia, which has almost 90° placement of the torso to the operating table, can be difficult and might require ultrasound-guided access [11]. Bart “flank-free” modified supine position was introduced to address these aforementioned difficulties. Its 15° tilt of the torso enables easy percutaneous fluoroscopy-guided access. It provides more space to place and dilate multiple tracts due to the neutral positioning of the kidney. Moreover, the intrarenal pressure is reduced due to the relatively horizontal tract, which also allows for the fragments to be washed out easily. This position is also similar to the original RIRS position and allows for an easy transition to ECIRS when needed [11]. 

The significance of our study is that to the best of our knowledge, our study is only the 2nd study using the Barts “flank-free” modified supine position. Our results are encouraging and comparable to the results of previously published cohorts with prone, Valdivia, complete supine, and the Barts modified Valdivia positions. The evaluation of postoperative residual stones with NCCT constitutes an important strength of our study.

Our study has some limitations. First of all, this was not a randomized study and all operations were not performed by the same surgeon. The study is retrospective, the number of cases is relatively low and may not be sufficient to identify significant differences. 

## 5. Conclusion

Our retrospective study suggests that in experienced hands, supine and prone PCNL appear to be equivalent in terms of stone-free rate and complications, and that supine PCNL is associated with a shorter operation time. The Barts “flank-free” modified supine position is an effective method that can be used safely. The results of our study are comparable with the results of previously published cohorts with the supine position. Broader randomized controlled trials are needed to strengthen our conclusions.

## Informed consent

Patient’s approval was taken. Ethical Approval received (Institutional Review Board approval number: 26379996/58).
